# Effects of Age and Self-Performance on Memory for the Actions of Other People

**DOI:** 10.1093/geroni/igab046.2622

**Published:** 2021-12-17

**Authors:** Alan Kersten, Julie Earles, Jennifer Brymer

**Affiliations:** 1 Florida Atlantic University, Boca Raton, Florida, United States; 2 Florida Atlantic University, Jupiter, Florida, United States

## Abstract

This research tested whether performing an action themselves leads young and older adults to have difficulty remembering which of a number of other people had performed that same action. It also tested whether observing another person perform an action leads to false memory for self-performance of that action. Young adults and healthy older adults 62 to 88 years of age viewed videos of actors performing actions. After viewing some of the actions, participants were instructed to perform those same actions themselves. Participants were tested one week later on their memory for their own actions and for the actions of the actors in the videos. Older adults were more strongly influenced by self-performance than were young adults when asked whether the actor in a test item had performed the same action previously. Young adults performed better than older adults at discriminating the correct and incorrect actors in the videos, although both groups showed reduced discrimination for actions that they had also performed themselves. The two groups were equally likely to falsely remember having performed an action that had only appeared in the videos, but young adults were better able than older adults to correctly identify the actions that they had in fact performed. Older adults thus have greater difficulty than young adults at distinguishing self-performed actions from actions performed by other people. This suggests the existence of common representations for the actions of oneself and others that must be bound to identity information to specify the correct source of the actions.

